# Scalable multiplexed machine learning gas sensor chips for food classification

**DOI:** 10.1126/sciadv.aec7965

**Published:** 2026-06-17

**Authors:** Carla Bassil, Kichul Lee, Xun Liao, Divya Krishnan, Yifei Zhan, Theodorus Jonathan Wijaya, Edward Hester, Minhyun Kim, Il-Doo Kim, Inkyu Park, Ali Javey

**Affiliations:** ^1^ Department of Electrical Engineering and Computer Sciences, University of California, Berkeley, CA, USA.; ^2^ Materials Sciences Division, Lawrence Berkeley National Laboratory, Berkeley, CA, USA.; ^3^ Berkeley Sensor and Actuator Center, University of California, Berkeley, CA, USA.; ^4^ Department of Mechanical Engineering, KAIST, Daejeon 34141, Republic of Korea.; ^5^ Department of Materials Science and Engineering, KAIST, Daejeon 34141, Republic of Korea.; ^6^ Kavli Energy NanoScience Institute, Berkeley, CA, USA.

## Abstract

Multiplexed gas sensor arrays combined with machine learning have unlocked previously inaccessible applications for scent-based sensing. Current platforms are limited by overlapping sensing materials with similar compositions, leading to highly correlated responses, or multistep deposition processes that hinder scalability. In this work, we developed a 16-element monolithic chip with fully distinct sensing layers, enabling a truly heterogeneous array. The system consists of highly sensitive carbon nanotube field effect transistors that are functionalized through a single-step microdispensing method compatible with automated pipetting systems. The resulting chip produces characteristic signal patterns in response to object-specific scent profiles and, when combined with machine learning algorithms, can perform automated object identification. We demonstrate the classification of 16 different objects, including food spoilage and nut allergens, with a 92.6% overall prediction accuracy.

## Teaser

A heterogeneous multiplexed gas sensor chip using microdispensed CNT-FET arrays for machine learning–based scent classification.

## INTRODUCTION

Scent is composed of complex mixtures of gas molecules including volatile organic compounds (VOCs), a class of chemicals that easily evaporate at room temperature (*1*, *2*). Gas profiles can inform us of the identity or presence of objects and their status. For example, most food products can be identified by their scent, and, over time, that scent profile evolves because of the volatiles released from bacterial metabolization and cellular processes (*3*–*5*). Capturing and making use of these gas composition data are heavily aided by large, multiplexed sensor arrays combined with machine learning (ML) (*6*, *7*). Currently, most single-chip gas detection systems rely on only 2 to 10 different sensors, and integrating multiple devices fabricated on separate chips introduces wiring complexity and bulky form factors (*8*–*10*). High-throughput evaporation methods have allowed for larger arrays; however, neighboring sensors share similar materials, resulting in overlapping responses (*11*, *12*). Further, most studies have relied on metal oxide semiconductors (MOSs) as the gas-sensitive layer, which typically requires high-temperature operation, restricting the platform to heat-tolerant materials (*13*–*16*). Achieving robust classification accuracy for a wide range of applications requires both many sensors and the use of wholly distinct sensing materials to generate diverse, nonoverlapping signal patterns.

Here, we address this problem using carbon nanotube (CNT) field-effect transistors (FETs) in which the base device structure is lithographically fabricated before the deposition of gas-sensitive layers. CNTs provide a high surface area–to–volume ratio, lending themselves to excellent room-temperature sensitivity and enabling the integration of polymers and other materials that would otherwise be incompatible at elevated temperatures (*17*–*19*). Since these solution-processable agents are restricted to the final device layer, microdispensing techniques can be used for facile deposition in a single step, yielding a truly heterogeneous and scalable sensor array. We combine this platform with convolutional neural networks (CNNs) to perform real-object identification on everyday foods. In particular, we highlight our ML-scalable CNT-based Electronic-nose Technology (ML-SCENT) can directly contribute to preventing foodborne illnesses and life-threatening allergic reactions by detecting food spoilage and allergens, addressing issues of broad societal importance.

## RESULTS

### Functionalized CNT-FET arrays for ML-based food classification

**Fig. 1.**
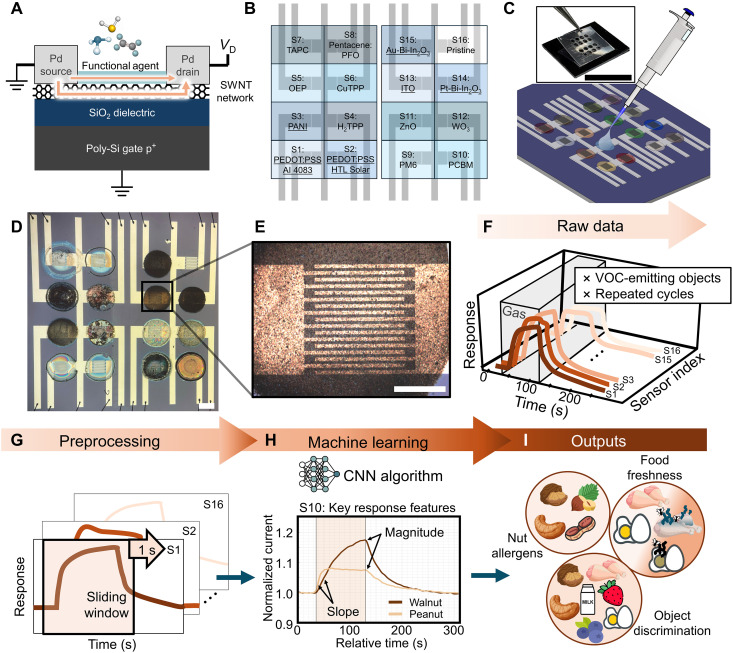
Overview of this study. (**A**) Single-device cross-sectional structure with arrows depicting possible current pathways. SWNT, single-walled carbon nanotube. (**B**) Functionalization material map for full 16-sensor array with conductive materials underlined (table S1). (**C**) Functionalization material deposition method via drop casting. Inset: Single-sided adhesive mask. Scale bar, 1.5 cm (**D**) Optical image of sensor array. Scale bar, 750 μm. (**E**) Optical image of ITO functionalized sensor. Scale bar, 200 μm. (**F** to **I**) End-to-end demonstration of the ML-SCENT for food classification: (F) demonstration of raw sensor signal responses to a single-gas pulse exposure across all 16 channels; (G) demonstration of data preprocessing through data partitioning and sliding window; (H) example of processed data from walnut and peanut VOC exposures, highlighting key features for CNN model recognition; and (I) model classification and discrimination between inter- and intracategory food objects.

Multiplexed 4 × 4 gas sensor arrays are developed by fabricating CNT-FET devices with gas-sensitive materials deposited on top of the channel (Fig. 1A and fig. S1). Sixteen materials were downselected and individually optimized for solution processing techniques, following literature data mining of previously demonstrated VOC-sensitive layers (Fig. 1B) (*20*–*24*). The array uses chemical and electronic diversity, incorporating four primary material classes: conductive polymers (*22*, *25*, *26*), porphyrins (*24*, *27*), organic semiconductors (*28*–*31*), and metal oxides (*32*–*34*). These film selections cause the variations observed in the *I*_D_ versus *V*_G_ characteristics across the array (fig. S2). This deliberate heterogeneity allows each sensor to interact differently to the same gas molecules, eliciting distinct response signals. The diversity in sensing behavior, rather than specificity toward any single analyte, serves as the informational foundation for ML-assisted object discrimination. This broad chemical coverage is required because identification of everyday foods involves decoding complex mixtures of gases that no single sensor material can distinguish. A series of solvents, concentrations, and drying temperatures were tested to refine film uniformity and reduce coffee-ring effects over the sensing area. The materials were microdispensed in one step over a custom laser-cut, single-sided adhesive mask, enabling a scalable process that can be easily automated using robotic pipetting systems (Fig. 1C). Figure 1D depicts an optical image of the full 7.5 mm–by–7.5 mm chip following solvent evaporation and mask removal, showing zero leakage of functional agents between adjacent sensors. We highlight indium tin oxide (ITO) dispersion in Fig. 1E, depicting homogeneous coverage of the film over the entire device channel.

We recorded sensor response by measuring the change in current through each FET over time while grounding the gate (*V*_G_ = 0 V) and applying a small 500-mV bias (with the exception of S11, which was operated at *V*_DS_ = 1 V to achieve a measurable baseline current) between the source and drain electrodes (fig. S3). The current pathway through the device depends on the conductivity of the functional agent (Fig. 1A and table S1). With nonconductive films, current travels exclusively through the CNT network, allowing the functional agent to serve as a semiselective membrane for charge transfer between gases and the channel. In the case of conductive films, current passes through both the CNT network and functional agent. Thus, the sensing mechanism of the device becomes more complex, as competing interactions can exist between the two layers. Gas molecules can physically adsorb onto the surface of functional agents and CNTs or chemically react to previously adsorbed species such as O_2_ or H_2_O. The result is a donation or withdrawal of electrons from the device channel (*35*). This temporary doping mechanism induces a shift in the threshold voltage (*V*_TH_) of the device, which is observed as an increase or decrease in current over time, aided by FET signal amplification (Fig. 1F) (*36*). Since each device has distinct transfer characteristics (fig. S2), this shift will lead to different degrees of current change. Devices operating in the subthreshold regime at *V*_G_ = 0 V will experience an exponential change in current, while devices in the linear regime will have a more moderate change. The mechanism of charge transfer–induced gas response for CNT based FETs is well established (*37*). However, as scent profiles can contain hundreds to thousands of different molecules, it is unfeasible to discern exactly which adsorption processes are at play. Luckily, these mechanistic details are not critical for object classification due to the aid of ML implementation.

ML-SCENT was exposed to 16 different objects including various fruits, nuts, and spoiled dairy and meat products. These food items produce a volatile landscape ranging from fruity esters and monoterpenes to oxidative aldehydes, heterocyclic pyrazines, and sulfur-containing compounds, underscoring that a simple sensor array cannot effectively discriminate this extensive chemical diversity (*38*–*41*). The data were collected using a multiplexer, which cycled through all 16 devices at a rate of 0.25 Hz (Fig. 1F and fig. S3). Target gases were exposed for 95 s with a 185-s recovery period between pulses. Following data collection, preprocessing in the form of normalizing, tagging, and slicing was implemented to prepare the dataset for ML (Fig. 1G). The data were then used to train a CNN, which recognizes key response features such as slope and magnitude (Fig. 1H) that inform us of differences in analyte adsorption and reaction potential. The ML model was lastly tested in its ability to discriminate between different inter- and intracategory food objects (Fig. 1I).

### Gas performance of ML-SCENT platform

ML-SCENT was tested for durability and repeatability over the course of 2 days using the walnut VOC scent profile (fig. S4A). Figure S4B depicts four pulsed exposures to 5 g of freshly chopped walnuts taken at 15.5-hour intervals, overlaid and normalized to the first-pulse baseline. The sensors show uniformity in both response magnitude and kinetics, indicating that these chips can be operated continuously for several days without requiring recalibration.

To characterize concentration dependency, we used varying quantities of chopped walnuts (0.05, 0.5, and 5 g) as a proxy for increasing VOC headspace concentration. Figure S5A illustrates the dynamic sensor responses across these conditions, while fig. S5B highlights the logarithmic relationship between sample mass and signal magnitude at the 120-s mark. While sensors S3 (PANI) and S13 (ITO) exhibited negligible sensitivity to these specific VOCs, the remaining 14 sensors showed distinct, concentration-dependent responses with signal magnitude scaling predictably with object quantity.

All real-food objects are expected to naturally release moisture, which becomes part of the overall gaseous headspace mixture. Hence, we characterized the effect of pure humidity to understand response trends and moisture susceptibility (fig. S6). We found that S2 (PEDOT:PSS HTL Solar) was the most reactive to moisture, while S3, S8, and S13 (PANI, pentacene, and ITO, respectively) were least affected. Porous materials such as S4 to S6 and S14 to S15 (porphyrin and Bi-In_2_O_3_ variants) displayed a higher humidity tolerance than S16 (pristine CNT), likely a result of physically blocking water vapor diffusion into the CNT matrix. Across all sensors, an initial “spike” was observed in the response profile. This can be attributed to the accumulation of water vapor in the headspace of the Erlenmeyer flask, to which the sensor is momentarily exposed to before reaching a lower steady-state response by the 120-s mark. A similar, albeit less pronounced, effect was observed in some food samples, reflecting the natural buildup of VOCs and moisture between pulses. This behavior highlights the dynamic nature of the headspace composition but does not impede the ability of the ML model to distinguish between complex mixtures. Ultimately, the amount of moisture released from food can vary considerably depending on storage conditions and the degree of spoilage. Hence, more comprehensive experimental testing and model training specifically addressing humidity effects across a wider range of food conditions will be necessary in future work.

As food spoils, it is known to release increased levels of amines, sulfides, and other VOCs (fig. S7) (*42*). Naturally, we characterized the effect of NH_3_ on our sensing layers (fig. S8A). We observed results consistent with the *n*- or *p*-type characteristics and *I*_D_-*V*_G_ profiles of the sensors (table S1 and fig. S2). To benchmark our system, we compared one of our devices, S12 (WO_3_), against several VOC sensors reported in the literature (fig. S8B). We found that ML-SCENT falls within sensitivity ranges typical of other established platforms (*43*–*53*).

**Fig. 2.**
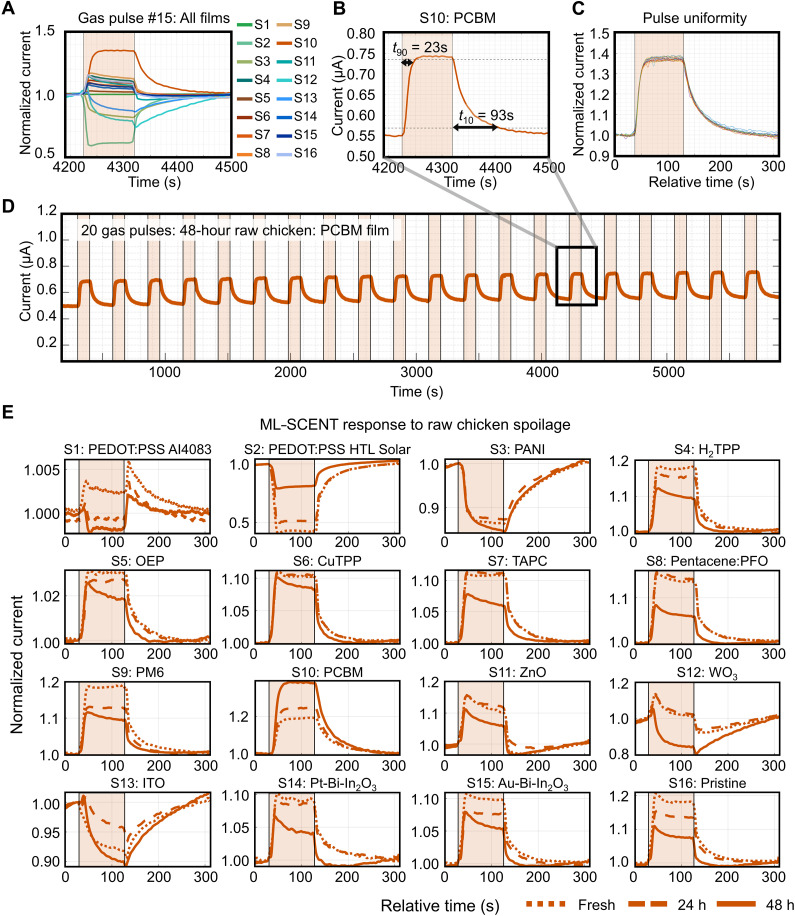
Responses highlighting sensor performance upon exposure to spoiling raw chicken. (**A**) Gas response from all 16 sensors to a single headspace exposure of 15 g of 48-hour (h) spoiled raw chicken. Gas exposure is depicted by a transparent orange box. (**B**) Individual PCBM functionalized sensor response with evaluated *t*_90_ and *t*_10_. (**C**) Nineteen repeated 48-hour spoiled raw chicken gas pulses partitioned and plotted in a single graph to demonstrate response uniformity to repeated cycles (first pulse removed to mitigate initialization effects). (**D**) Raw data from S10 (PCBM) in response to 20 pulsed exposures to the target gas. (**E**) Raw data from pulse #15 of all sensors in response to fresh, 24-hour, and 48-hour spoiled chicken for comparison.

Figure 2 (A to D) highlights sensor performance in response to 48-hour spoiled raw chicken (15 g). Functionalization resulted in different exposure profiles as seen among the 16 devices in Fig. 2A. Figure 2B takes a closer look at sensor number 10, which was functionalized with *n*-type polymer PCBM ([6,6]-phenyl C61 butyric acid methyl ester). We observed a quick *t*_90_ at 23 s, which reflects the time required to reach 90% of the saturated response. The recovery is measured by *t*_10_ at 93 s, defined as the time required to return to 10% of the initial baseline value. While the response and recovery times may vary depending on the target analyte, both were quick compared to many other benchmarks that can take tens of minutes to hours (*54*, *55*). It should be especially noted that, in contrast to typical MOS devices, no thermal or light assisted resetting of the device was required for recovery. The entire measurement was conducted at room temperature using dry air as the carrier gas. An automated gas exposure system was used to pass air over the scent-emitting object (fig. S9). In this way, VOCs from the object headspace were captured and swept into the device chamber. The chip was exposed to each object’s scent 20 times. The first pulse was removed to mitigate inconsistencies due to system initialization. The latter 19 pulses were split up, normalized to the baseline, and presented in a single plot to check for uniformity (Fig. 2, C and D, and fig. S10).

Figure 2E compares individual sensor responses to a single pulse of fresh, 24-hour, and 48-hour aged raw chicken stored in a standard Erlenmeyer flask at room temperature. We found that in our experimental setup, humidity levels are not significantly altered as the chicken spoils, indicating that increasing concentrations of reducing gases are driving the trends in sensor response (fig. S11). The combined gas chromatography-mass spectroscopy and humidity datasets suggest that the gaseous headspace of fresh chicken is dominated by water vapor (figs. S7 and S11). Hence, the fresh chicken response of most sensors closely resembles that of pure humidity (fig. S6B). As chicken spoils, the NH_3_ response trends can be used to inform how increasing levels of reducing gases should affect the signal. It is evident that the increase/reduction of response magnitude upon exposure to more spoiled samples follows the direction of ammonia-induced conductivity changes seen in fig. S8A. For instance, the response profile for S16 continually decreases in magnitude as the chicken sample spoils, consistent with the conductivity reduction observed in the presence of ammonia (Fig. 2E and fig. S8A). Hence, the sensor array effectively captures the combined influence of moisture and spoilage VOCs through relative signal shifts across timestamps. The material diversity and resultant selectivity enable a versatile platform capable of resolving these complex, competing chemical interactions.

### Data preparation and ML implementation

**Fig. 3.**
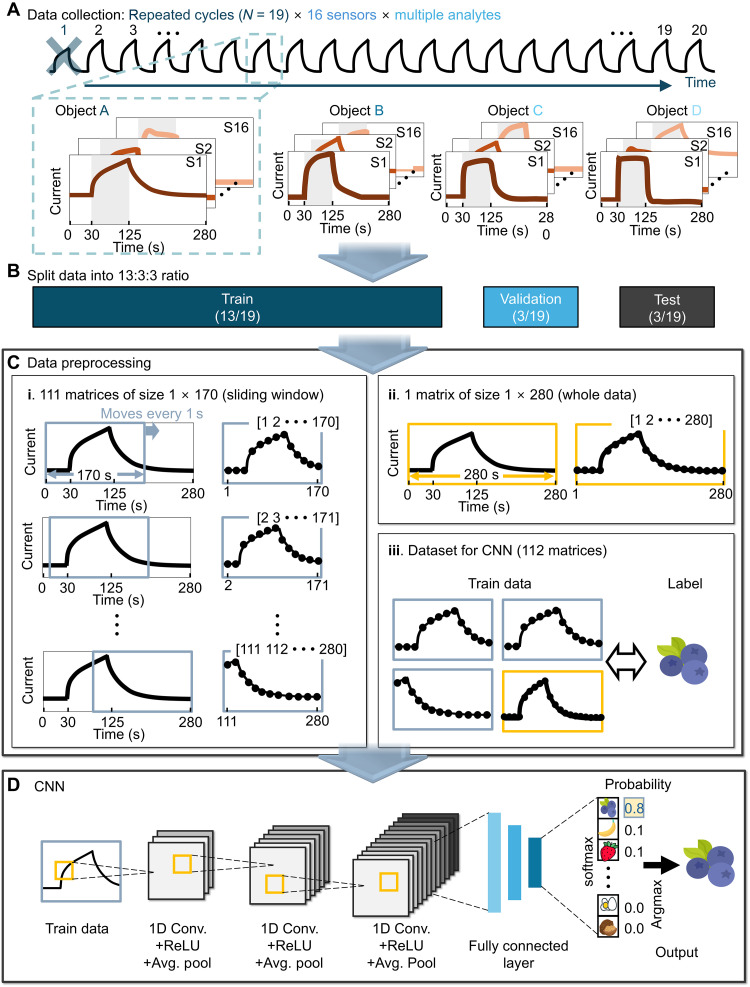
Workflow of data preprocessing and model training in this study. (**A**) Data collection using 16 sensors simultaneously, repeated for 20 cycles across multiple objects (first pulse removed to mitigate initialization effects). (**B**) Data splitting into training, validation, and test sets in a 13:3:3 ratio after excluding the first cycle. (**C**) Data preprocessing by applying a sliding window to the 280-s 1D signals; both 170- and 280-s windows were fed into the model. (**D**) Prediction of the object with the highest probability after passing through three 1D convolutional layers, followed by an FC layer.

The time-series data underwent preprocessing before introduction to the ML model. Since the data was originally collected at a rate of 0.25 Hz, it was resampled at 1-s intervals using interpolation that preserved the transient shape of the original data. The raw measurements were segmented into 280-s datasets, each consisting of 30-s baseline, 95-s gas exposure, and 155-s recovery (Fig. 3A). The recovery period exceeded the *t*_10_ of all sensing films, minimizing carryover effects between measurements. Each dataset was normalized to the baseline at 30 s, and the first pulse was omitted to remove initialization artifacts. The 19 remaining datasets were then randomly split into a 13:3:3 ratio, separated for training, validation, and testing, respectively (Fig. 3B). For clarity and ease of understanding, from Fig. 3C onward, we present the preprocessing procedure using only one sensor and a single-gas exposure cycle as an example.

Within these partitioned data buckets, a sliding window of 170 s with 1-s shifts was applied to generate 111 additional datasets, corresponding to all possible 170-s windows within the 280-s cycle (Fig. 3C-i). In addition, the entire 280-s cycle was treated as one dataset, resulting in a total of 112 datasets per gas pulse (Fig. 3C-ii and iii). Using a window size smaller than the full dataset allows the CNN to focus specifically on local and transient patterns, enabling the model to effectively capture subtle, time-sensitive features. This approach also significantly increases the number of available data samples and their diversity, exposing the model to a richer variety of signal variations and enhancing its ability to classify new data (*8*). These datasets were then assigned a ground-truth label corresponding to the object of exposure using one-hot encoding. It should be noted that the sliding window datasets were contained within the initial train, validate, and test buckets such that no data leakage occurred that could lead to model overfitting.

Last, the preprocessed data along with ground-truth labels were fed into a CNN, a class of deep learning models and a powerful tool for temporal data. CNN uses convolution filters that slide over adjacent data points and calculate a representative value for the group, highlighting important features while retaining local relationships. This property gives CNN an advantage for sensor data analysis, where transient responses are indicative of stiction coefficients and gas adsorption rates characteristic to each sensing material and gas pair (*56*–*58*).

Similar to how a color image is composed of three RGB (red-green-blue) channels, the time-series data were concatenated to form 16 distinct channels that shared the same temporal axis. These one-dimensional (1D) datasets were then fed into a CNN specifically designed for multichannel data (Fig. 3D). The model architecture was composed of three convolutional blocks, followed by a flattening layer and a fully connected (FC) layer. The first convolutional layer, using 32 filters with a kernel size of 4, transformed the 16 input channels into 32 new feature maps. The data were then passed through a rectified linear unit (ReLU) activation function to introduce nonlinearity. The subsequent average pooling layer then downsampled the temporal dimension by a factor of 2, reducing the input length from 280 to 140. This process of feature extraction and downsampling was repeated twice more, with the number of filters doubling at each step. The data’s dimensions were transformed from (280, 16) to (140, 32), then to (70, 64), and lastly to (35, 128). The output from the last convolutional block, with a shape of (35, 128), was flattened into a single 1D vector of size 4480 (35 × 128) before being fed into the FC layer with an output node for each object label. Then, a softmax activation function was applied to produce a probability distribution over the labels. The final prediction was obtained by applying the argmax operation to this distribution, selecting the label with the highest probability. For training, the model used the categorical cross-entropy loss function to quantify the discrepancy between the predicted and true one-hot encoded labels. This loss was minimized through an iterative process over multiple epochs using the Adam optimizer.

### Inter- and intracategory object classification results

Here, we demonstrate our ML-SCENT for use in food safety. According to the Centers for Disease Control and Prevention, approximately 48 million Americans suffer from foodborne illnesses each year (*59*). Foodborne bacteria emit characteristic gases as they metabolize organic products for which gas sensors can be developed and trained to detect. In this study, an ML-SCENT chip was exposed to the gaseous headspace of 15 g of raw chicken, 24 g of boiled egg, and 75 ml of whole milk in their safe-to-eat state and then subsequently 1 and 2 days after being left out at room temperature. A second chip was prepared to further investigate the utility of our platform to discriminate between 3 g of nut varieties (walnut, hazelnut, cashew, and peanut), which are listed among the Food and Drug Administration’s top 9 food allergen categories in the United States. To our knowledge, this is the first demonstration of a gas sensor array being used as a method to detect the presence of different nut allergens. Last, to evaluate intercategorical discrimination using the same chip we added 2 g of freeze-dried fruits (blueberries, strawberries, and bananas) to the object repository.

**Fig. 4.**
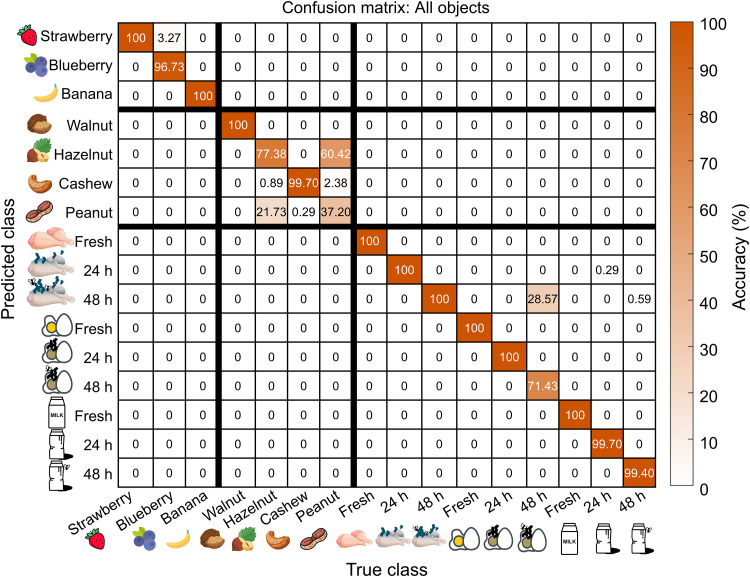
Confusion matrix results from CNN model trained on all food spoilage products, nuts, and fruit. The model achieved an overall accuracy of 92.6%, with the majority of errors arising from intracategory misclassifications.

We trained a single model on sensor response data collected from all tested objects (Fig. 2E and figs. S12 and S13). This model, demonstrated in Fig. 4, resulted in a 92.6% overall accuracy, which was calculated by dividing the number of correct predictions by the total number of predictions in the dataset. The largest confusion arose between hazelnut and peanut VOCs, indicating some cross-over between nut odorants or shared dominant compounds that elicit a similar sensor response. The prediction mistakes between 48-hour spoiled boiled egg and raw chicken again suggest similar gaseous compound development between the two foods, likely due to increased levels of amines, sulfides, and thiols. The confusion matrix results demonstrate that our platform can near-perfectly discriminate between intercategory objects, with little to no misclassification arising between the fruit or nut food categories, which were detected using the same ML-SCENT chip. However, the aforementioned prediction mistakes also suggest that intracategory object discrimination is a more challenging task. This discrimination can be aided by increased training dataset size, better tailored sensing material selection, or improvements in the CNN model.

### Application-specific models for food spoilage and nut allergen detection

**Fig. 5.**
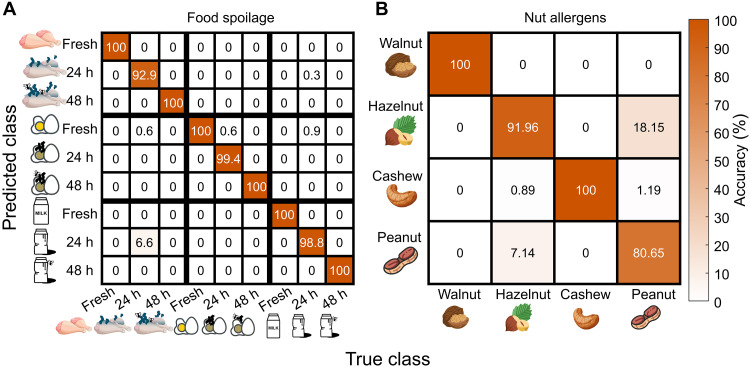
Confusion matrices for two separate applications trained on different ML models. (**A**) Confusion matrix for a CNN model trained on all food spoilage data. (**B**) Confusion matrix for a separate CNN model trained only on nut allergens.

We address the issue of intracategorical confusion by training application-specific models for the food spoilage and nut categories. Using the same food spoilage data as before, Fig. 5A depicts the prediction accuracy for raw chicken, boiled egg, and whole milk. Here, we can see the model effectively predicts which food and spoilage state each gas profile represents, with an overall accuracy of 99.0%. When the training set is restricted to just food spoilage objects, the model can properly extract and highlight the features seen in Fig. 2E and fig. S12, resulting in near-perfect classification.

Fig. 5B depicts the prediction accuracy between each nut allergen, with an overall success rate of 93.2%. The hazelnut and peanut success rates increased from 77.4 to 92.0% and 37.2 to 80.7%, respectively, compared to Fig. 4. This application-specific model again shows a significant improvement in the platform’s classification ability simply by restricting the dataset to fewer ground-truth classes. In addition to improved outcomes, more focused, smaller models reduce computation time, power, and cost resources.

### Optimizing sensor count

**Fig. 6.**
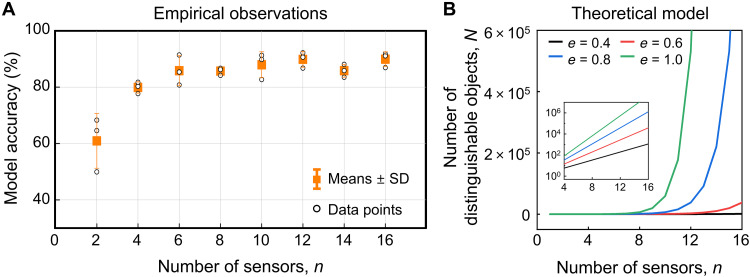
Empirical and theoretical assessment of model accuracy and number of identifiable objects as a function of the number of sensors in an array. (**A**) Empirical data for overall model accuracy to 16 VOC-emitting objects using only a subset of 2, 4, 6, 8, 10, 12, 14, or 16 sensors. For each subset size, the model was trained on three different random groupings of sensors drawn from the full array, and error bars indicate the means ± SD across these runs. (**B**) Theoretical relationship between the number of distinguishable objects (*N*) and the number of sensors (*n*), given *N* = *k^en^* (example, *k* = 3), where *e* represents sensor efficiency. Curves show how sensor efficiency, which reflects redundancy or overlap among gas sensors, influences the exponential growth of 𝑁 as the number of sensors, 𝑛, increases.

Classification accuracy is expected to rise as additional sensors are added to the array. Here, we randomly selected a subset of sensors to test how sensor count affects model prediction accuracy across all 16 objects. Three random sensor groupings were used for each subset, and their SDs are depicted in Fig. 6A. Performance rose from 49.9 to 92.6% as additional sensors were included. As expected, the increasing trend eventually saturated, and there was a point at which adding more sensors no longer had a significant effect on the outcome.

To move beyond purely empirical observations, we sought to frame these results with a simple mathematical perspective. We suppose that an array contains *n* sensors, each capable of generating *k* distinct response patterns (for simplicity, *k* = 3; positive, negative, or no response). The theoretical number of unique signal combinations that the array can produce is *k^n^*. If we further assume that each sensor-response combination corresponds one to one with a distinguishable gas, then it follows that the number of gases the system can discriminate grows exponentially with the number of sensors. Of course, real systems deviate from this idealization, and some gases may elicit very similar signals, leading to ambiguity. As emphasized in this study, sensor redundancy arising from fabrication-induced similarity leads to near-clones with overlapping responses, limiting the effective array size. To capture this, we define an effective sensor number, $n_{\text{eff}}=e\cdot n$ (with efficiency factor *e*) and the corresponding combinations $N=k^{n_{\text{eff}}}$. Figure 6B shows this relationship for *e* = 0.4, 0.6, 0.8, and 1.0. The inset illustrates that higher efficiency (*e* = 1.0) enables exponentially greater classification capacity than lower efficiency (*e* = 0.6) or equivalently achieves the same performance with nearly half as many sensors, highlighting the need to minimize redundancy for a powerful electronic-nose (e-nose) system.

## DISCUSSION

In summary, we present the successful implementation of 16 distinctly functionalized sensors integrated monolithically into a single array using a highly scalable microdispensing technique to perform food object classification with CNN algorithms. As discussed in this work, the ideal case of distinguishing up to *k^n^* objects requires maximizing the number of unique sensors by minimizing overlap in composition and response. In our current implementation, all devices share CNT as the common channel material, yet the microdispensed functional layers provide substantial differentiation. As a result, the array achieved reasonable accuracy in classifying 16 objects with as few as *n* = 4 sensors, and performance improved further when scaled up to *n* = 16 (Fig. 6A). Looking ahead, incorporating alternative base materials could expand the effective diversity of responses, bringing the platform closer to the theoretical limit and enabling higher prediction accuracy. Our platform offers ample opportunity for scale-up through additional sensing layers, expansion of the array pinout, and integration with automated pipetting systems. Beyond just FET-based platforms, we anticipate the incorporation of multimodal gas sensors as the next generation of e-nose devices, strengthening diversity and adaptability of the final platform for a richer set of applications.

## MATERIALS AND METHODS

### CNT-FET fabrication

A 90-nm SiO_2_/Si *p*^++^ wafer was lithographically patterned using S1818 photoresist (Kayaku Advanced Materials Inc., USA) to create sixteen 0.6 μm–by–0.4 μm rectangular openings for CNT deposition. Poly-l-lysine solution [0.1% (w/v) in H_2_O; Sigma-Aldrich] was first drop casted over this pattern. After 5 min, the solution was rinsed off with deionized (DI) water and dried using a nitrogen (N_2_) gun. A 99% semiconductor-enriched single-walled CNT solution (NanoIntegris) was then deposited for 30 s, followed by DI water rinse and N_2_ spray drying. The photoresist was subsequently removed using Remover PG (Kayaku Advanced Materials Inc., USA), and the wafer was baked at 200°C in ambient air to assist in the removal of surfactant residues from the CNT aqueous solution. Interdigitated electrodes with 10-μm channel length and 0.5/45-nm Ti/Pd-thick contacts were patterned using standard metal lift-off procedures. The *I*_D_-*V*_G_ characteristics of each device contained within the array were tested to ensure functionality (fig. S1).

### Functional material preparation

Functionalized materials for 15 sensors (excluding the pristine CNT matrix on sensor 16) were optimized for drop-casting compatibility.

1) Sensors 1 and 2: PEDOT:PSS HTL Solar (1:2.5, w/w) and AI4083 (1:6, w/w) were purchased from Ossila and prepared as a 25% (v/v) H_2_O solution. The solution was briefly vortexed and dispensed immediately. The relative ratios of PEDOT and PSS in these two formulations allow for differences in conductivity, acidity, and film composition.

2) Sensor 3: PANI (weight-average molecular weight > 15,000) powder was purchased from Sigma-Aldrich and prepared in a dimethyl sulfoxide (DMSO) solution (50 mg/ml) stirred at 60°C overnight, followed by filtration through a 5-μm pore size.

3) Sensors 4 to 6: 5,10,15,20-(Tetraphenyl)porphyrin (H_2_TPP), 2,3,7,8,12,13,17,18-(octaethyl)porphyrin (OEP), and copper(II) 5,10,15,20-(tetraphenyl)porphyrin (CuTPP) were purchased from PorphyChem and dissolved in a 4.4 mM dichloromethane solution. All porphyrins were stirred in solvent at 50°C for 4 hours.

4) Sensor 7: 4,4′-Cyclohexylidenebis[*N*,*N*-bis(4-methylphenyl) benzenamine] (TAPC) was purchased from Osilla, dissolved in a chlorobenzene solution (10 mg/ml), and stirred at 50°C for 2 hours.

5) Sensor 8: Poly(9,9-di-*n*-octylfluorenyl-2,7-diyl) (PFO) and pentacene were purchased from Luminescence Technology Corporation and dissolved together in a 1,2-dichlorobenzene (oDCB) solution (15 and 0.3 mg/ml), respectively. Pentacene:PFO was stirred in solvent at 150°C overnight.

6) Sensor 9: Poly[(2,6-(4,8-bis(5-(2-ethylhexyl)-4-fluorothiophen-2-yl)-benzo[1,2-b:4,5-b’]dithiophene))-alt-(5,5-(1’,3’-di-2-thienyl-5’,7’-bis(2-ethylhexyl)benzo[1’,2’-c:4’,5’-c’]dithiophene-4,8-dione))] (PM6) was purchased from 1-Material, prepared in a chloroform solution (0.9 mg/ml), and stirred at 50°C for 4 hours.

7) Sensor 10: PCBM was purchased from Solaris Chem and dissolved in a oDCB solution (90 mg/ml) stirred at 60°C overnight, followed by filtration through a 5-μm pore size.

8) Sensors 11 and 12: Zinc oxide (ZnO) nanoparticle ink (0.9% concentration dispersion in 2-propanol) and tungsten oxide (WO_3_) nanoparticle ink (2.5 wt % in 2-propanol) were purchased from Sigma-Aldrich and used as is.

9) Sensor 13: ITO nanopowder (<50-nm particle size) was purchased from Sigma-Aldrich and dissolved in a DMSO solution (1 mg/100 μl), stirred at 50°C for 4 hours, and immediately vortexed before use.

10) Sensors 14 and 15: Pt-Bi-In_2_O_3_ and Au-Bi-In_2_O_3_ nanofibers were obtained by modifying a previously reported protocol (*60*). Bi-doped In_2_O_3_ nanofibers were synthesized by electrospinning and calcination processes. They then underwent incipient-wetness impregnation with 1% of Pt or Au precursors contained in DI water solutions. The mixtures were dried and calcinated at 300°C (ramping rate, 10°C/min) for 10 min. The products were dispersed in a DMSO solution (1 mg/100 μl), stirred at 60°C overnight, and immediately vortexed before use. The use of different metal nanoparticles diversifies catalytic and adsorption properties of the sensors (*61*).

All solvents were purchased from Sigma-Aldrich. Characteristic contact angles over the SiO_2_ substrate interface are included in fig. S14.

### Deposition of the functionalized materials

A one-sided Lensguard 7568 adhesive film (Nitto Corp.) was custom laser-cut with a pattern of 1-mm circular openings over every sensor location. This film was then aligned to the completed CNT-FET array. The 15 films were then deposited via drop casting over each individual opening and dried at room temperature unless otherwise specified. The chip was placed on a hot plate and heated to 70°C for PCBM deposition and 40°C for ZnO and WO_3_ deposition to control drying rates. After all solvents had dried, the chip was heated at 70°C for 8 min, after which the Lensguard film was peeled away. Film thickness information was measured by means of Veeco Dektak 6M profilometry (fig. S15). *I*_D_ vs *V*_G_ measurements were performed on the fully functionalized chip to ensure viability of all devices following deposition (fig. S2).

### Gas test setup

Dry air (AI BR-K cylinder, Linde Gas & Equipment Inc.) was used as the carrier gas in all experiments. Three mass flow controllers (MFCs), purchased from MKS, were used to route the carrier gas through clean lines or through an Erlenmeyer flask containing a VOC emitting object at a flow rate of 500 standard cubic centimeters per minute (fig. S9). An Arduino Opta was programmed to control MFC valve open/close operations. To mitigate start-up transients and stabilize drift, the sensor chip was operated overnight for ~10 hours with clean air flow before testing. Quantities of food placed inside the flask are as follows: 3 g of nuts, 2 g of dried fruit, 15 g of raw chicken, 24 g of boiled egg, and 75 ml of whole milk. For food spoilage experiments (chicken, egg, and milk), the initial fresh measurement was taken, after which the flask was removed from the system and set aside in ambient, room-temperature conditions to age. During these aging periods and in between all food types, a clean flask was installed, and the system lines were purged for 10 min with the carrier gas to flush out any remaining VOCs and prevent cross-contamination. After each spoilage interval, the original flask containing the aged food was returned to the system for measurement.

### Sensor measurement scheme

The sensor array was mounted onto a 68-pin chip carrier (Evergreen SemiConductor Materials Inc.). This chip was placed inside a custom-built gas chamber (fig. S16) with air-tight electrical routings. The MAX336EPI+ multiplexer chip from Analog Devices, controlled by an Arduino Uno, was used to cycle through all 16 sensors at a rate of 0.25 Hz. The B2912 Keysight source measurement unit was used to supply a constant drain-source bias of 500 mV (with the exception of S11, which was operated at *V*_DS_ = 1 V to achieve a measurable baseline current) to the chip and record current over time. A custom MATLAB (MathWorks, USA) code was written to synchronize the data collection scheme with MFC switching.

### Data processing and ML environment

The preprocessing of datasets, training, and evaluation of the ML models were implemented in PyTorch, an open-source deep learning library developed by Meta (USA), together with other open-source Python libraries for data handling, ML, and visualization.
