# Supplementary material for: Scalable multiplexed machine learning gas sensor chips for food classification
Source: Sci Adv. 2026 Jun 17;12(25):eaec7965. doi: 10.1126/sciadv.aec7965 (PMC13274616; doi:10.1126/sciadv.aec7965)
Supplement: Supplementary file 1 — Figs. S1 to S16 Table S1 [file sciadv.aec7965_sm.pdf]

Science Advances
AAAS

Supplementary Materials for

Scalable multiplexed machine learning gas sensor chips for food classification

Carla Bassil *et al.*

Corresponding author: Inkyu Park, inkyu@kaist.ac.kr; Ali Javey, ajavey@berkeley.edu

Sci. Adv. **12**, eaec7965 (2026)
DOI: 10.1126/sciadv.aec7965

This PDF file includes:

Figs. S1 to S16
Table S1

Fig. S1.

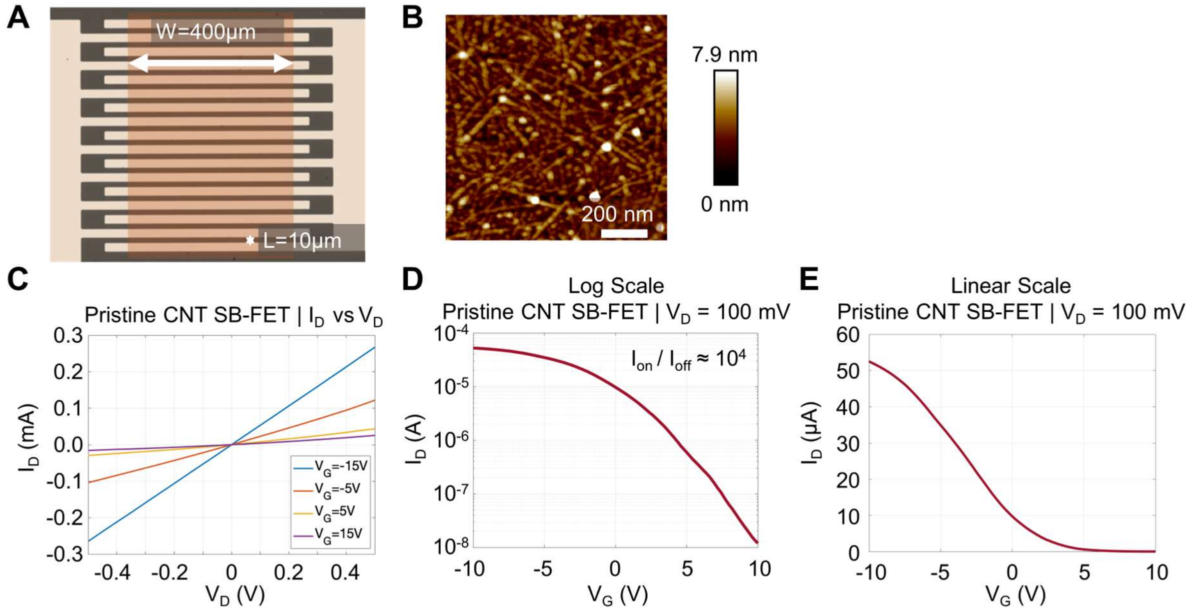

Pristine CNT-FET characteristics. (**A**) Optical image of CNT-FET electrodes with channel dimensions. (**B**) Atomic force microscope (AFM) scan of CNT matrix in the channel. (**C**) I_D-V_D curve of CNT-FET for V_G = -15, -5, 0, 5, and 15 V. (**D**) Log scale I_D-V_G curve for CNT-FET at V_D = 100 mV with V_G swept from -10 V to 10 V in 200 mV increments. (**E**) Linear scale I_D-V_G curve for CNT-FET at V_D = 100 mV with V_G swept from -10 V to 10 V in 200 mV increments. SB stands for Schottky barrier.

Fig. S2.

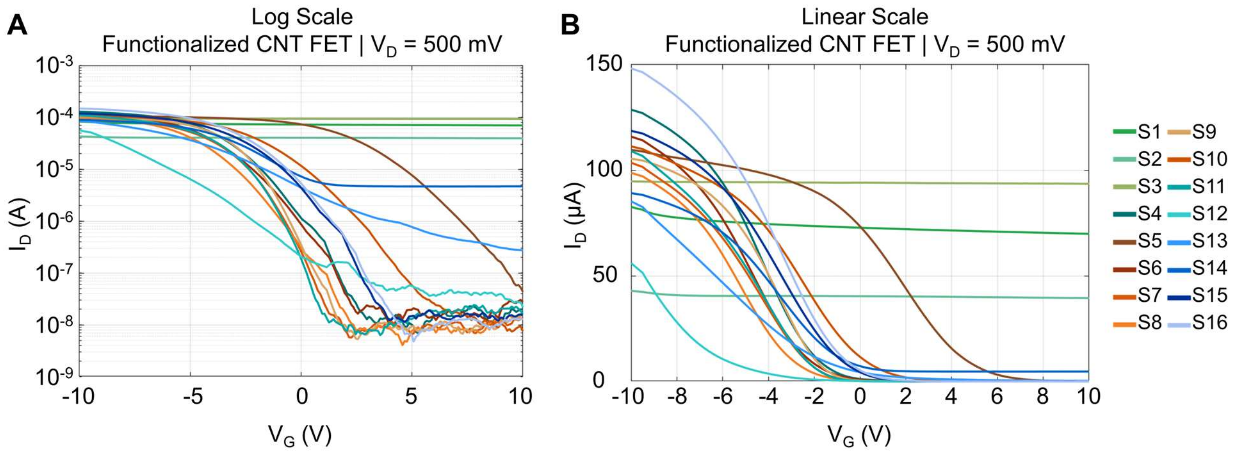

I_D-V_G graph of fully functionalized sensor array with V_D = 500 mV and gate voltage swept from -10 V to +10 V in 200 mV increments. (**A**) Plotted in log scale. (**B**) Plotted in linear scale.

Fig. S3.

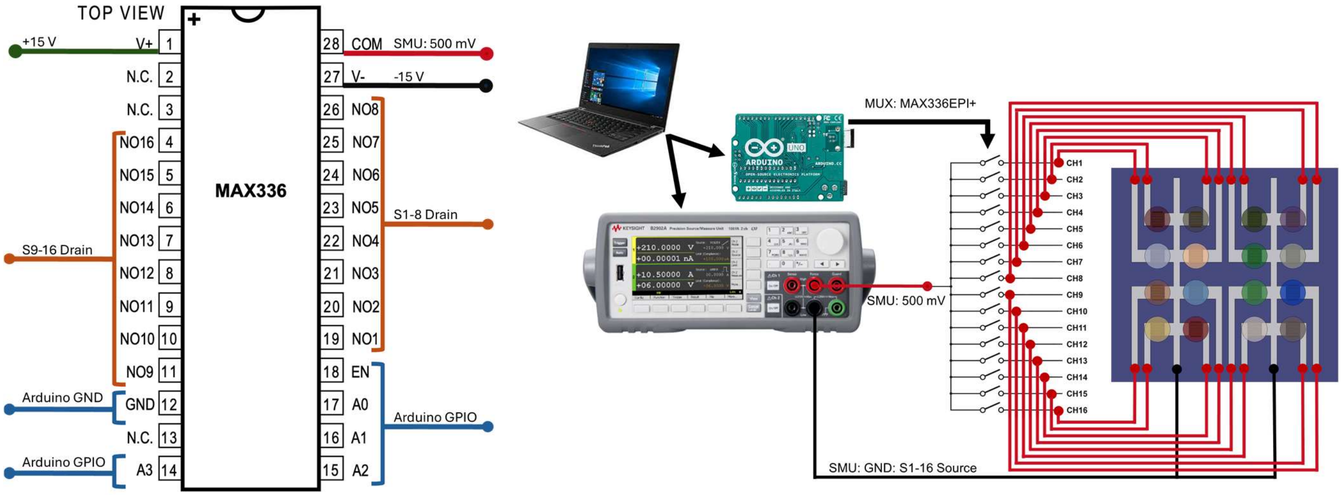

Detailed depiction of electrical connections to ML–SCENT chip. The drain electrodes of all 16 FET devices were connected to 16 individual multiplexer channels. One source electrode was shared between 8 FET devices and connected to ground along with the back gate of the chip. The SMU provided the drain-source bias voltage of 500 mV (with the exception of S11, which was operated at V_{DS} = 1 V to achieve a measurable baseline current) through the MUX. The MUX switching was controlled using an Arduino Uno, and the SMU measurement was automated using a custom written MATLAB code. Here, N.C. represents no connection.

Fig. S4.

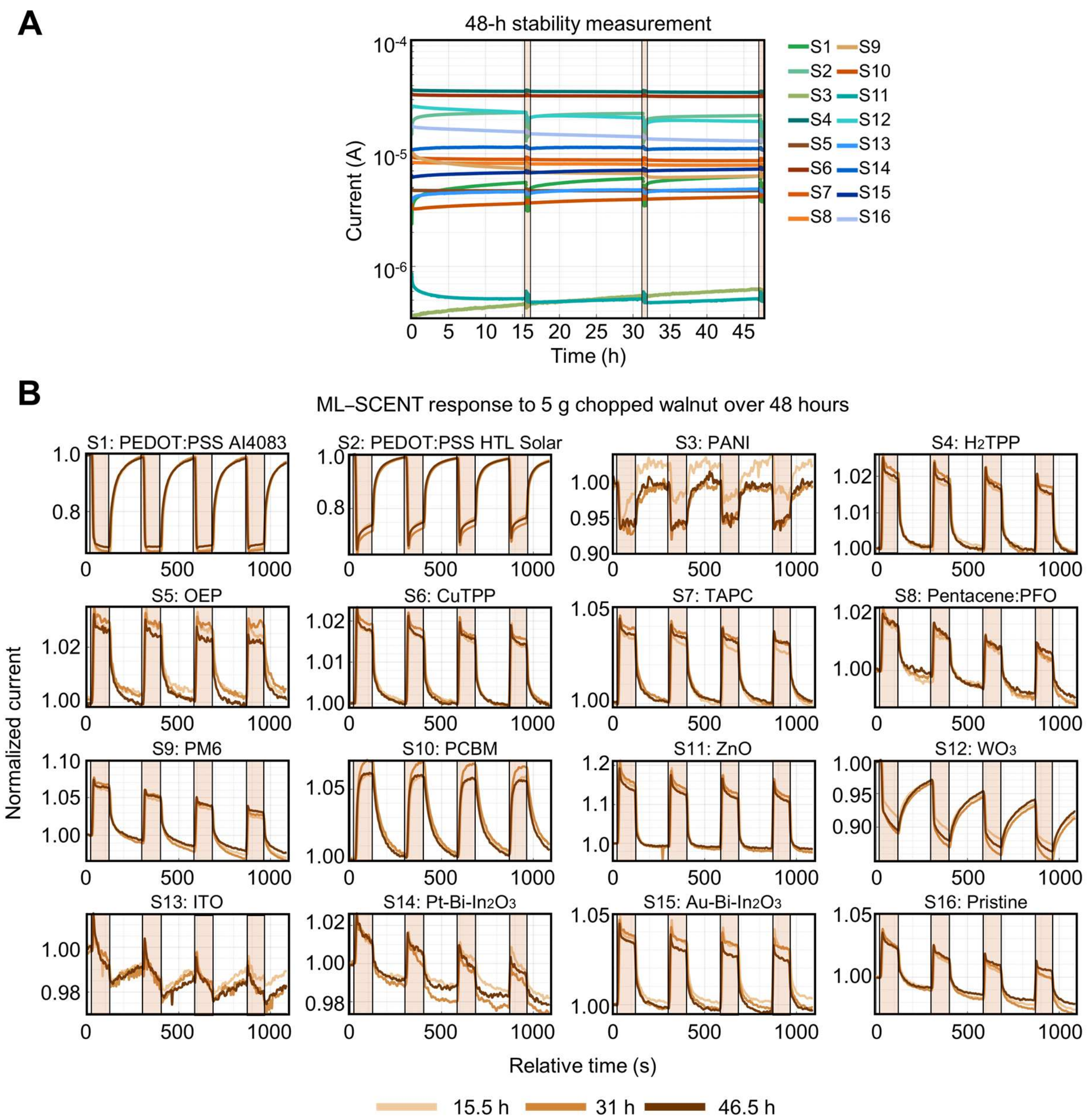

Sensor stability over two-day long measurement. (**A**) A two-day continuous stability measurement was conducted with 5 g of chopped walnut exposed every 15.5 hours following the same flow and timing conditions as described in the *Data preparation and machine learning implementation* section of the main manuscript. The entire measurement is depicted with walnut pulses highlighted by an orange box. (**B**) Sensor responses to each timepoint of four pulsed walnut exposures, overlaid and normalized to the first-pulse baseline of each experiment. Repeatability and uniformity indicate sensor durability over the measured timeline.

Fig. S5.

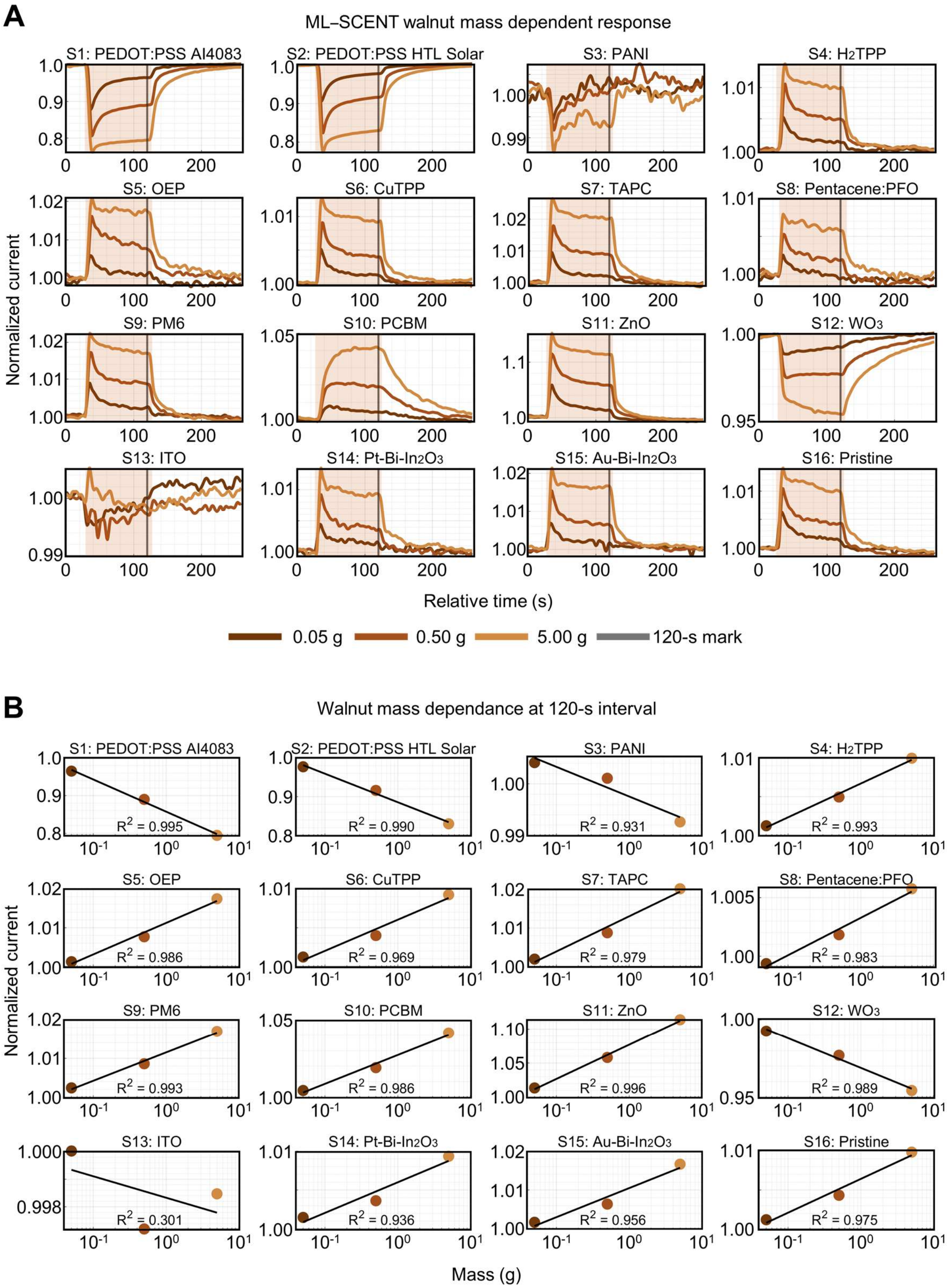

VOC concentration dependence. (**A**) ML-SCENT sensor responses upon exposure to the VOC headspace of 0.05 g, 0.5 g, and 5 g of chopped walnut. (**B**) Mass dependence of response magnitude at the120-s response time mark.

Fig. S6.

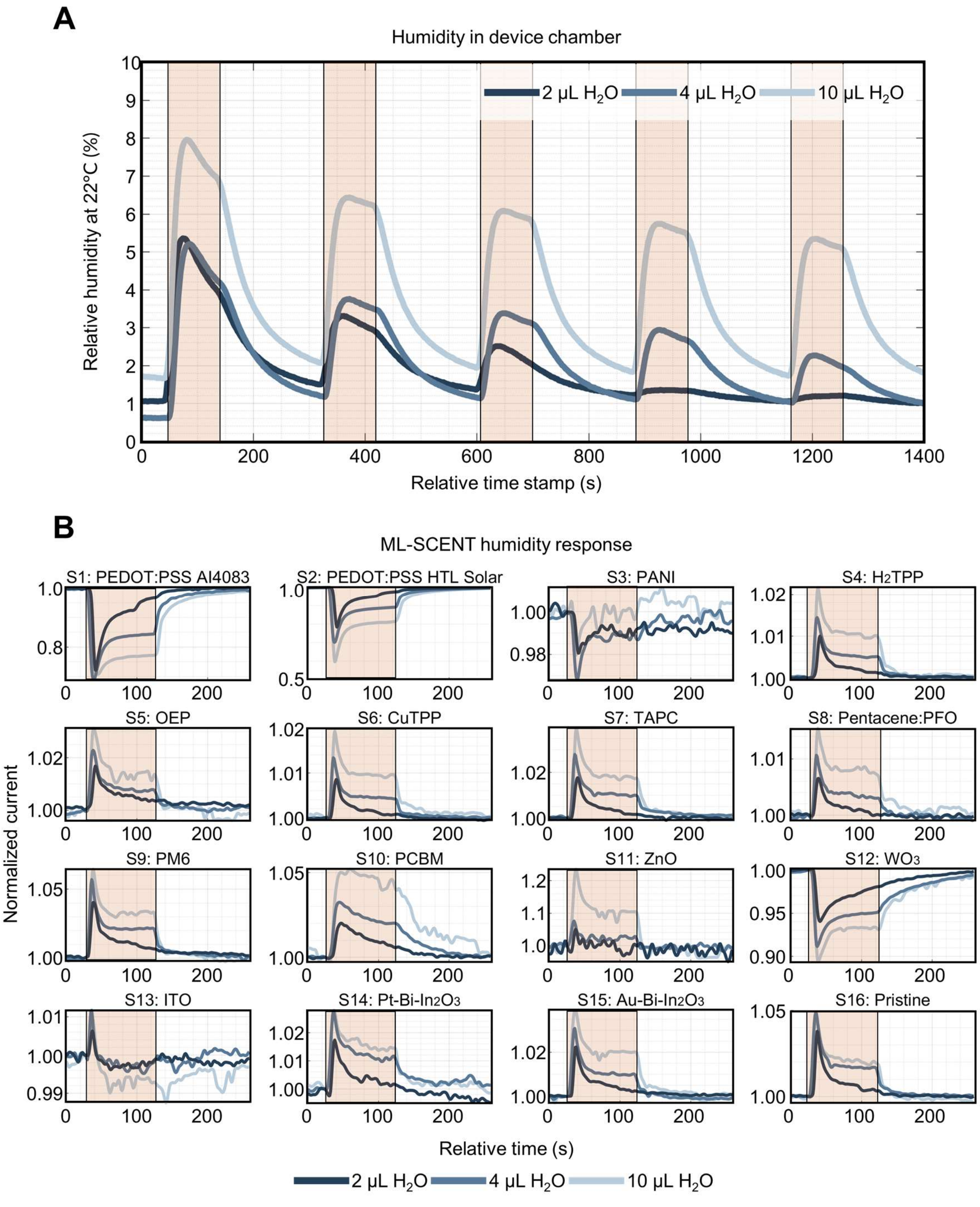

Humidity in the device chamber at 22℃. Three experiments were conducted in which 2 µL, 4 µL, and 10 µL droplets were placed inside the flask. The sensor was exposed to 5 pulses of each condition following the same flow and timing conditions as described in the *Data preparation and machine learning implementation* section of the main manuscript. (**A**) Depicts the relative humidity inside the device chamber during the experiment as recorded by BME688, Bosch commercial sensor. (**B**) ML-SCENT individual sensor responses to the 3rd pulse exposure of each of the water volume conditions.

Fig. S7.

A

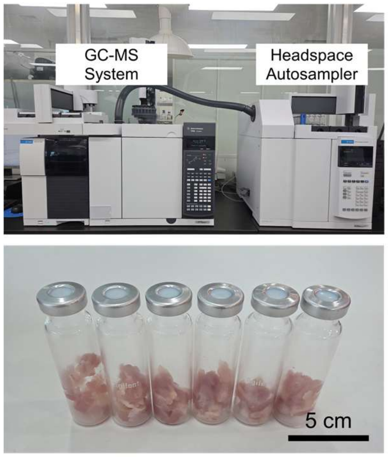

(1) Headspace Autosampler

Parameter	Condition
Instrument	Agilent 7697A Headspace Autosampler
Heating temp.	50°C
Equilibration time	20 min
Sample Volume	5 g

(2) GC-MS

Parameter	Condition
Instrument	Agilent 7890B GC / 5977B MS
Column	DB-WAX, 60 m × 250 μm, 0.25 μm
Carrier gas	He, 1 mL/min
Injection mode	Split, 10:1
Injection volume	1 mL
Inlet temperature	200 °C
Oven program	30°C (0 min) → 5°C/min → 200°C (5 min)
Ionization mode	Electron Ionization
Ion source temperature	230°C
Scan range	45 - 450 m/z
Solvent delay	0 min

B

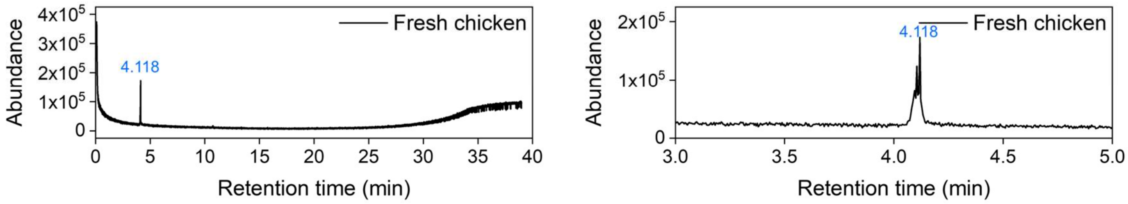

NIST Library search report

Pk #	RT	Area %	Library	Ref#	CAS#	Qual
1	4.118	100.00	D:\MassHunter/Library/NIST17.L			
			Dimethyl ether	107	000115-10-6	5
			Dimethyl ether	108	000115-10-6	5
			Ethanol	104	000064-17-5	4

C

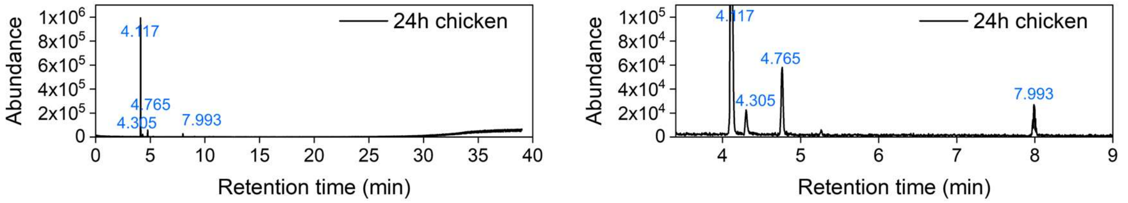

NIST Library search report

Pk #	RT	Area %	Library	Ref#	CAS#	Qual
1	4.117	83.79	D:\MassHunter/Library/NIST17.L			
			Dimethyl ether	108	000115-10-6	5
			Dimethyl ether	107	000115-10-6	5
			Ethanol	102	000064-17-5	4
2	4.305	3.54	D:\MassHunter/Library/NIST17.L			
			L-Alanine, 3-sulfo-	42445	000498-40-8	74
			N,N'-Nonamethylenebis[-S-3-aminopropyl thiosulfuric acid]	289463	035871-58-0	74
			Aminomethanesulfonic acid	6842	013881-91-9	74
3	4.765	8.43	D:\MassHunter/Library/NIST17.L			
			Methanethiol	123	000074-93-1	90
			Methanethiol	124	000074-93-1	83
			Methional	5239	003268-49-3	9
4	7.993	4.23	D:\MassHunter/Library/NIST17.L			
			Dimethyl ether	107	000115-10-6	9
			Ethanol	104	000064-17-5	7
			Ethanol	103	000064-17-5	7

D

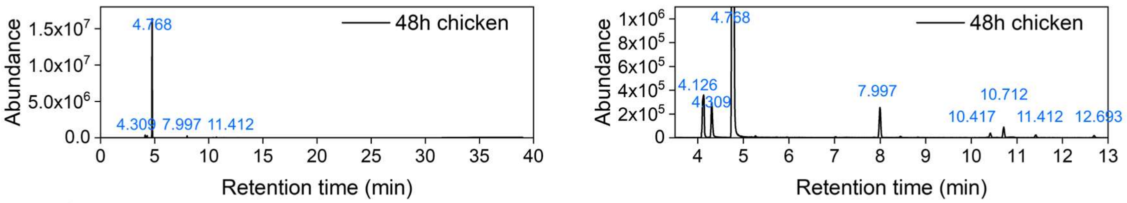

NIST Library search report

Pk #	RT	Area %	Library	Ref#	CAS#	Qual
1	4.126	2.60	D:\MassHunter/Library/NIST17.L			
			Ethene, fluoro-	101	000075-02-5	3
			Formic acid	110	000064-18-6	3
			Formic acid	111	000064-18-6	3
2	4.309	1.77	D:\MassHunter/Library/NIST17.L			
			Sulfur dioxide	409	007446-09-5	90
			Sulfur dioxide	410	007446-09-5	83
			Aminomethanesulfonic acid	6842	013881-91-9	74
3	4.768	93.12	D:\MassHunter/Library/NIST17.L			
			Methanethiol	123	000074-93-1	78
			Methanethiol	124	000074-93-1	78
			Methional	5239	003268-49-3	9
4	7.997	1.50	D:\MassHunter/Library/NIST17.L			
			Dimethyl ether	106	000115-10-6	9
			Dimethyl ether	107	000115-10-6	9
			Ethanol	104	000064-17-5	7
5	10.417	0.24	D:\MassHunter/Library/NIST17.L			
			2-Fluropropene	327	001184-60-7	83
			2-Fluropropene	328	001184-60-7	64
			Allyl fluoride	326	000818-92-8	40
6	10.712	0.51	D:\MassHunter/Library/NIST17.L			
			Methyl thioacetate	2532	001534-08-3	78
			3-Thietanol	2525	010304-16-2	78
			3-Thietanol	2526	010304-16-2	64
7	11.412	0.15	D:\MassHunter/Library/NIST17.L			
			Disulfide, dimethyl	2814	000624-92-0	94
			Disulfide, dimethyl	2811	000624-92-0	91
			Disulfide, dimethyl	2810	000624-92-0	91
8	12.693	0.12	D:\MassHunter/Library/NIST17.L			
			Propane, 2-methyl-1-(methylthio)-	5359	005008-69-5	50
			1-Pentanethiol	5333	000110-66-7	23
			D-Galactose, diethyl mercaptal, pentaacetate	295393	006935-10-0	12

Gas chromatography-mass spectroscopy (GC-MS) for spoiling chicken. (**A**) Left: overview of the GC-MS system integrated with a headspace autosampler and 5 g of raw chicken sample in a headspace vial. Right: table of headspace autosampler and GC-MS conditions. (**B-D**) Comparison of VOC profiles in chicken samples by decomposition time. (**B**) Fresh chicken. Minimal VOC generation with low peak intensities. (**C**) 24h-chicken. A significant increase in the overall peak magnitudes is observed, indicating the active generation of various VOCs, along with the emergence of sulfur-based gases such as L-alanine, 3-sulfo- and methanethiol. (**D**) 48h-chicken. Marked increase in overall VOC intensities, with methanethiol becoming the most dominant gas. Diverse sulfur compounds, including sulfur dioxide, methyl thioacetate, and dimethyl disulfide, are observed at advanced spoilage stages.

Fig. S8.

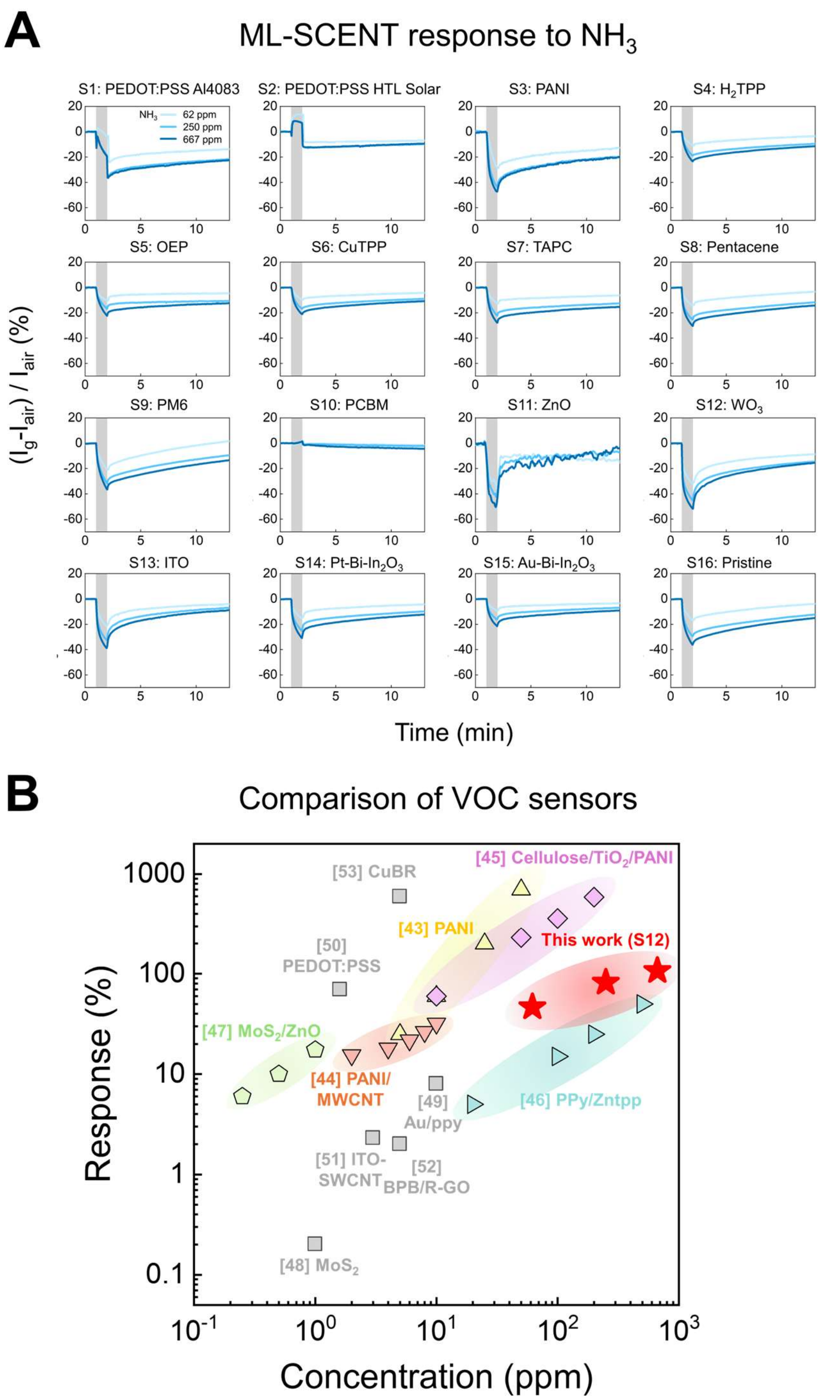

Characterization of ML-SCENT ammonia response (**A**) Response of 16 sensors to three different NH_3 concentrations (62, 250, and 667 ppm). The NH_3 gas was precisely delivered to the sensors from a cylinder tank using MFCs for accurate flow control and balanced with dry air. (**B**) Comparison of the CNT-FET gas sensor (S12: WO_3) in this study with other VOC gas sensing systems based on NH_3 testing for quantitative benchmarking. The response is defined as $(R_g-R_{air})/R_{air}$ or $(R_{air}-R_g)/R_g$.

Fig. S9.

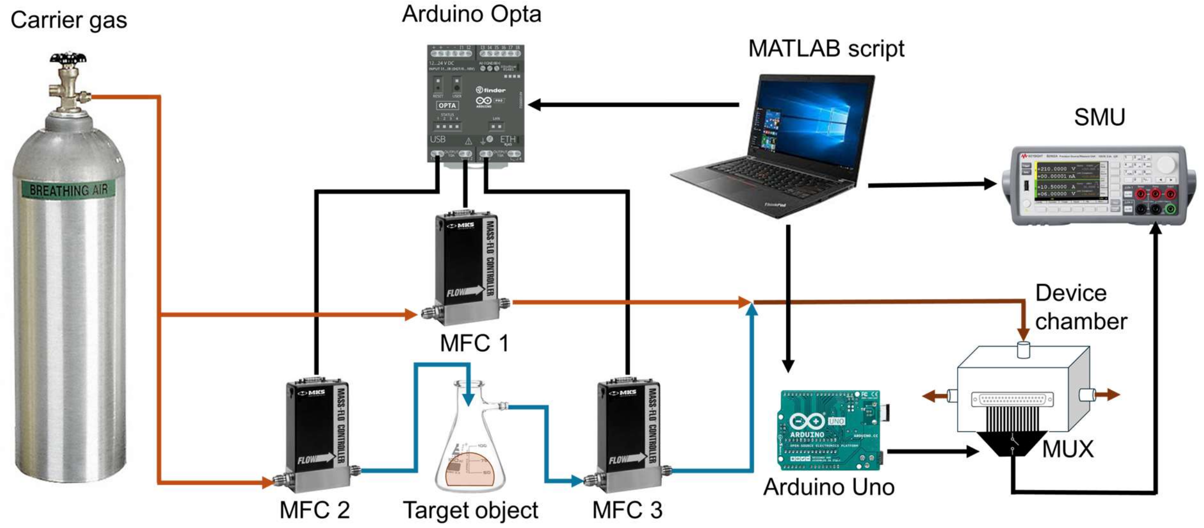

Experimental gas setup for this study. MFC 1 line was opened for baseline, recovery, and purge steps. MFCs 2 and 3 were opened for VOC gas exposure pulses and purging steps through an empty flask. Arduino Opta was programmed to provide valve open/close signals to the MFCs. The SMU was used to provide V_D bias to FETs and record the current over time. An Arduino Uno was programmed to control a multiplexer which sequentially cycled through all 16 sensors. MATLAB script was used to synchronize the MFC switching and data collection.

Fig. S10.

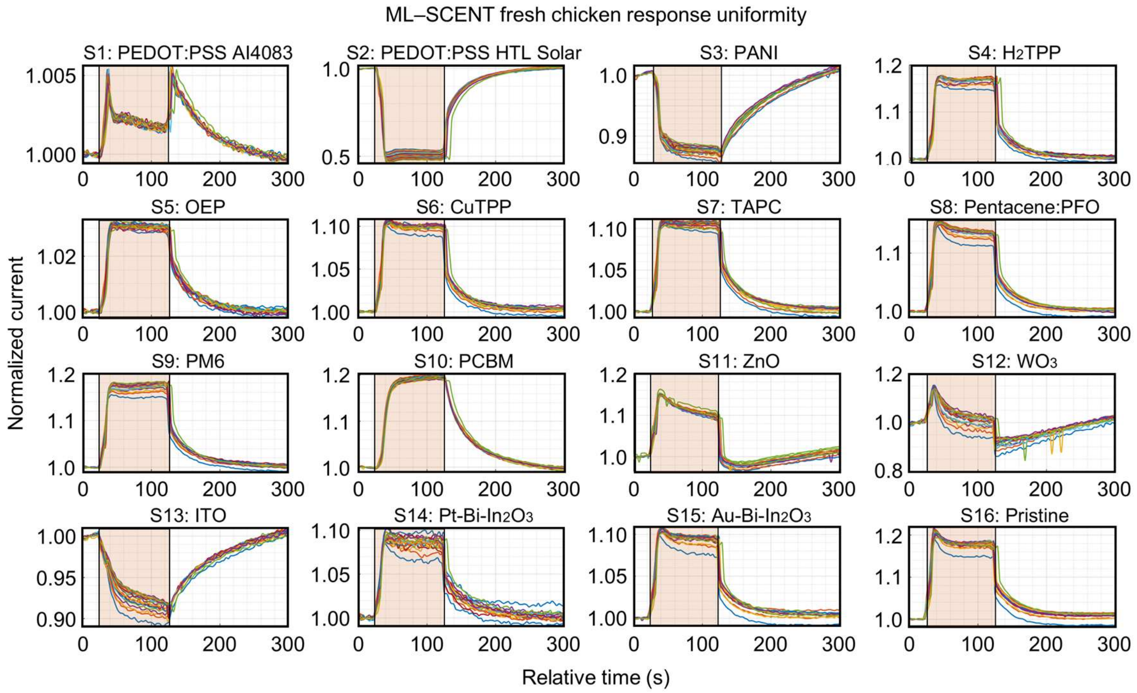

Response uniformity. Nineteen repeated gas pulses to 15 g of fresh raw chicken, partitioned and plotted in a single graph to demonstrate response uniformity to repeated cycles (first pulse removed to mitigate initialization effects).

Fig. S11.

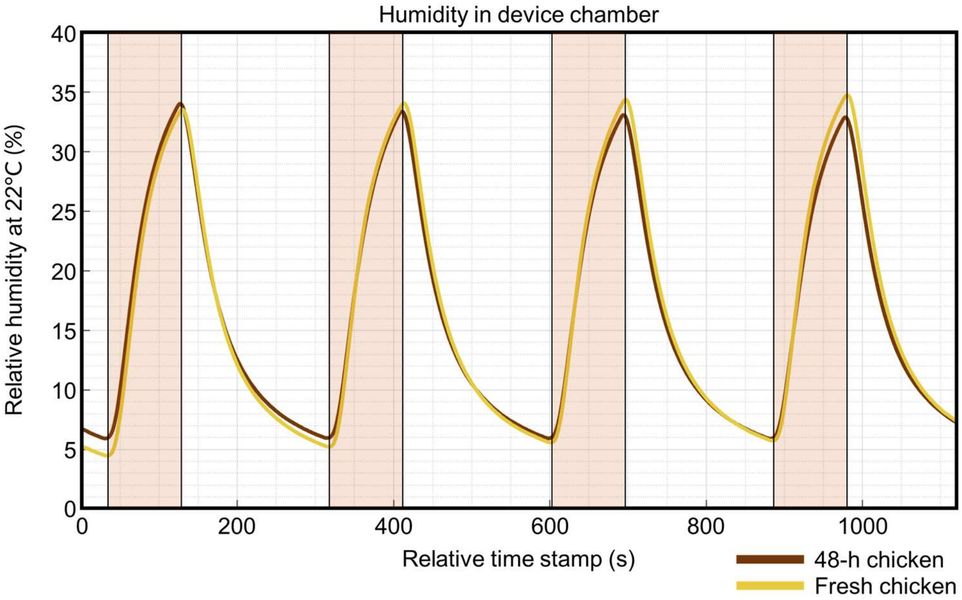

Humidity in the device chamber. Relative humidity (BME688, Bosch) at room temperature within the device chamber during four pulsed exposures to 5 g of fresh and 48-h spoiled chicken. No significant variation observed in humidity levels.

Fig. S12.

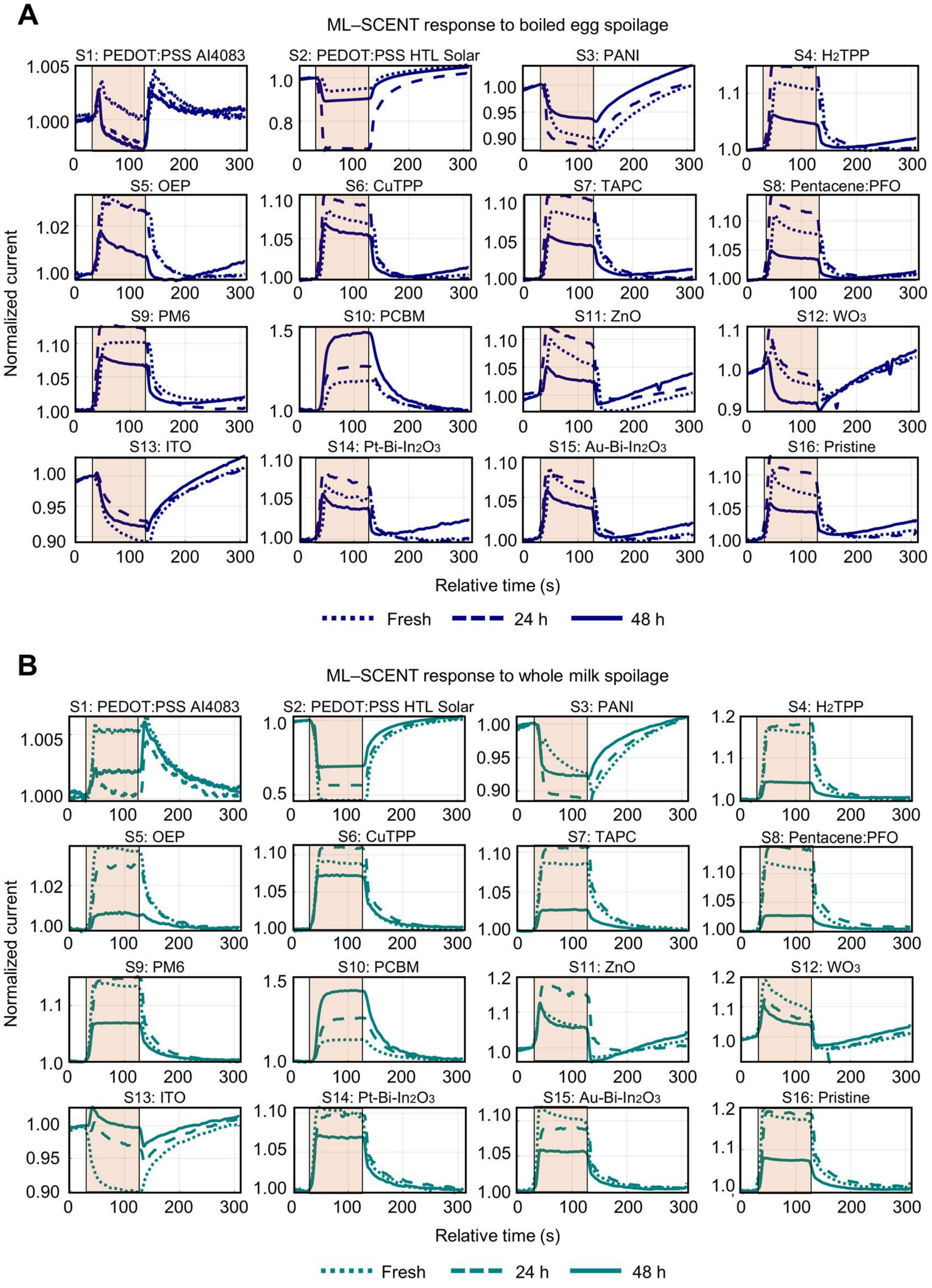

Raw data for exposure to spoiling boiled eggs and whole milk. (**A**) Raw data from pulse #15 of all sensors in response to fresh, 24 h, and 48 h spoiled boiled eggs (24 g) for comparison. (**B**) Raw data from pulse #15 of all sensors in response to fresh, 24 h, and 48 h spoiled milk (75 mL) for comparison.

Fig. S13.

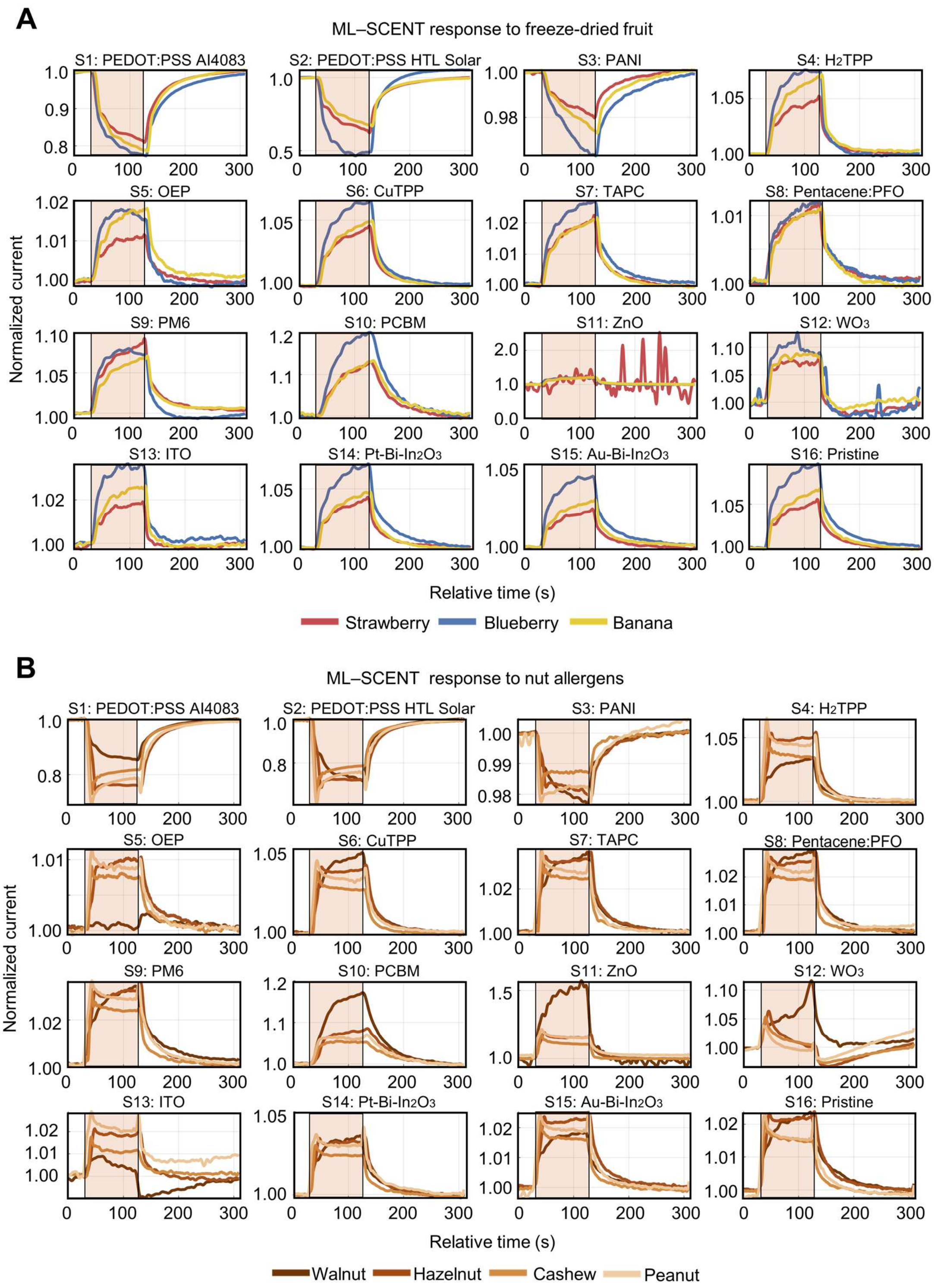

Raw data for exposure to fruits and nuts. (**A**) Raw data from pulse #5 of all sensors in response to freeze-dried fruit (2 g) for comparison. (**B**) Raw data from pulse #8 of all sensors in response to nut allergens (3 g) for comparison.

Fig. S14.

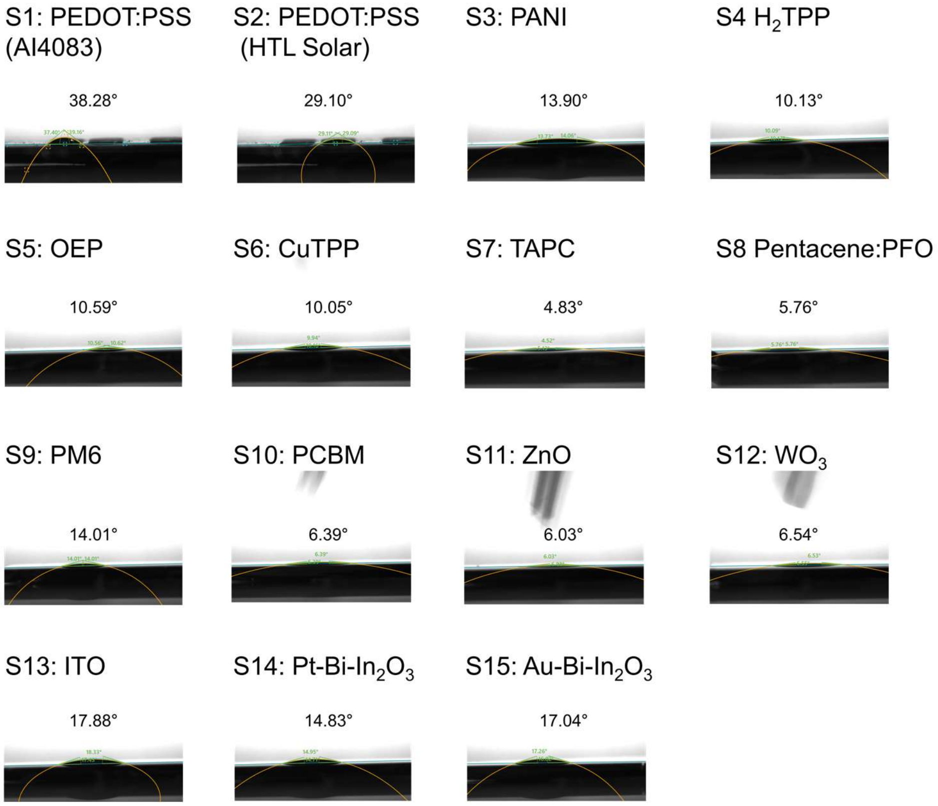

Contact angle characterization. Contact angle measurements of the 15 sensing materials dropped onto the 90 nm SiO_2/Si p++ substrate.

Fig. S15.

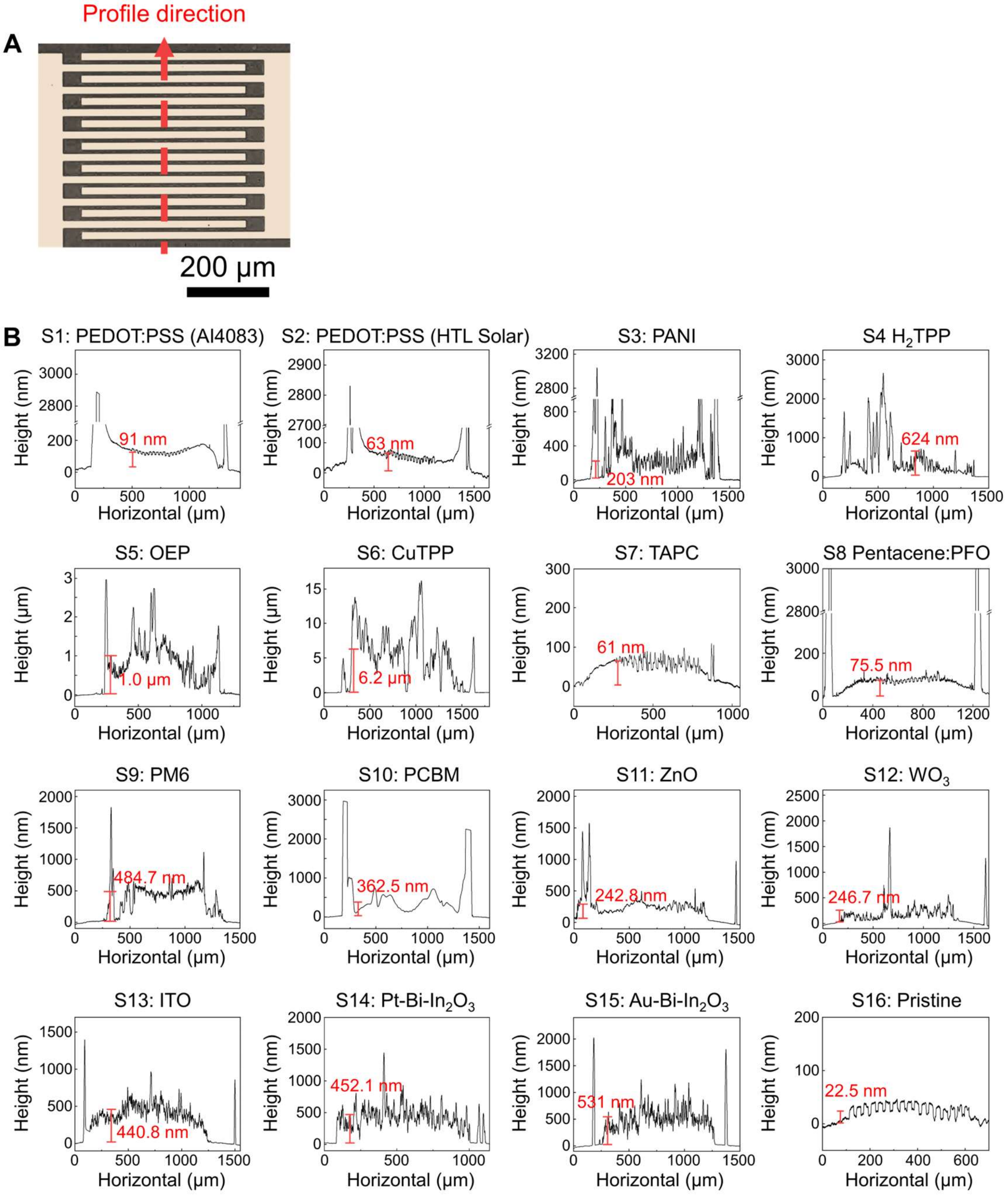

Thickness analysis of the 16 sensing materials via Veeco Dektak 6M profilometry. (**A**) Scanning path of the profilometer across the sensing layer. (**B**) Profilometry results for the sensing materials. All functional materials were deposited atop the interdigitated electrodes.

Fig. S16.

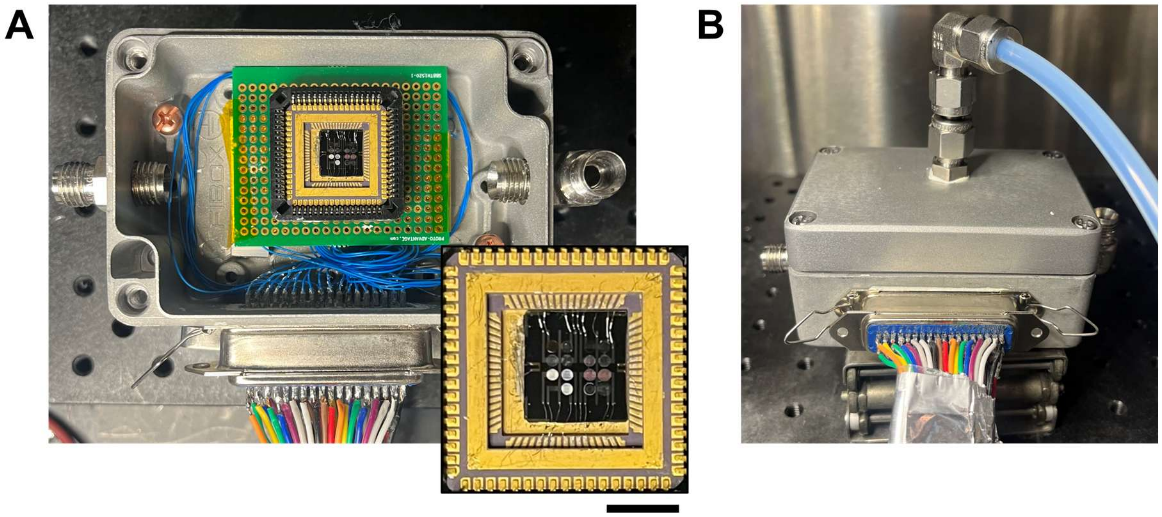

Photograph of the gas chamber employed in this study. (**A**) Sensor chip wirebonded to chip carrier and connected to D-Sub cable. Scale bar: 7.5 mm. (**B**) Air-tight closed chamber depicting gas inlet (top) and two outlets (sides).

Table S1.

List of all functionalization materials and electrical properties. Conductivity of each material was tested after drop casting over bare electrodes of the same dimensions and materials as in the CNT-FET devices. Biased at 500 mV with the gate floating, materials conducting current greater than 10 nA were labeled as conductive.

**Material**	**Type (N/P)**	**Conductivity (Y/N)**
Pristine CNT	P-type	Y
PEDOT:PSS AI 4083	P-type	Y
PEDOT:PSS HTL Solar	P-type	Y
PANI	P-type	Y
TAPC	P-type	N
Pentacene:PFO	P-type	N
PM6	P-type	N
PCBM	N-type	N
ZnO	N-type	N
WO_3	N-type	N
ITO	N-type	Y
Pt-Bi-In_2O_3	N-type	Y
Au-Bi-In_2O_3	N-type	Y
H_2TPP	N.A.	N
OEP	N.A.	N
CuTPP	N.A.	N

N.A.: Not applicable.
